# Harnessing artificial intelligence to decode the rhizosphere microbiome

**DOI:** 10.1016/j.abiote.2025.100005

**Published:** 2025-10-21

**Authors:** Juan Ma, Jiangfang Qiao, Yanyong Cao, Zeqiang Cheng

**Affiliations:** Institute of Cereal Crops, Henan Academy of Agricultural Sciences, Zhengzhou, 450002, China

**Keywords:** Artificial intelligence, Microbiome engineering, Rhizosphere microbiome, Microbiome-enabled genomic selection, Predictive modeling

## Abstract

The rhizosphere microbiome plays crucial roles in plant health by regulating nutrient cycling and enhancing stress resilience. However, due to its complexity, the rhizosphere microbiome is quite challenging to analyze using conventional approaches. Recent advances in artificial intelligence (AI) offer unprecedented opportunities to decipher intricate microbial interactions and leverage their potential for crop breeding. In this review, we assess AI methodologies derived from human microbiome studies that address foundational data challenges, including high dimensionality, compositionality, and sparsity. Next, we examine the uses of these methods for the functional prediction of microbial traits. We then shift our focus to the rhizosphere, exploring AI-driven approaches for predictive modeling of rhizosphere dynamics, integrating plant phenotypic and microbiome data, and designing synthetic microbial communities (SynComs). Finally, we discuss the major challenges and future prospects of using AI in rhizosphere microbiome research. Specifically, we propose an emerging AI paradigm that integrates complementary inside-out (hologenome-based genomic selection) and outside-in (SynCom design) strategies, powered by transformative technologies such as federated learning, large language models, digital twins, and autonomous AI agents. This review underscores the potential for AI to revolutionize microbiome science and crop improvement.

## 1 Introduction

The rhizosphere, the soil region enveloping a plant root and shaped by its biochemical activities, hosts a community of soil microbes known as the rhizosphere microbiome (rhizobiome). Together, the plant root, the rhizosphere soil, and the rhizobiome form a highly dynamic ecosystem that serves as the focal point for metabolic processes of the soil microbial community. This community is often referred to as the “second genome” of plants, as it plays crucial roles in plant nutrient acquisition, stress resilience, and growth promotion [1]. Heritable traits of the plant host were recently shown to actively shape the composition of the rhizosphere microbial community [2,3]. In unfavorable environments, roots in the rhizosphere actively recruit beneficial microbes, primarily by producing exudates containing sugars, amino acids, and secondary metabolites, with protective functions during drought, nutrient stress, and pathogen attack [[4], [5], [6]]. A notable example comes from maize (*Zea mays*), whose root-secreted flavones, such as apigenin and luteolin, enrich specific beneficial taxa such as Oxalobacteraceae, which in turn improve plant growth and nitrogen acquisition under nitrogen-deprived conditions [7]. This interaction represents a co-evolutionary partnership that is encapsulated in the hologenome concept, which describes the plant host and its associated microbiome as a unified functional entity, or holobiont.

The heritability of the plant's ability to shape its rhizosphere microbiome is a fundamental component of the hologenome concept. Quantitative trait locus mapping and genome-wide association studies have demonstrated that variation in the rhizosphere microbiome composition is associated with host genetics in crops such as maize, sorghum (*Sorghum bicolor*), eggplant (*Solanum melongena*), and tea (*Camellia sinensis*) [3,[8], [9], [10], [11], [12]]. Reported heritability estimates (broad-sense or narrow-sense) for these microbiome-associated traits typically range between 0.10 and 0.34, reinforcing the potential for microbiome-mediated crop improvement [[8], [9], [10], [11]]. This heritable capacity to shape the microbiome positions it as an important breeding target, effectively extending the plant's phenotype and offering novel approaches to crop improvement. Therefore, unraveling the molecular mechanisms underlying the crop-mediated regulation of the rhizosphere microbiome and their ecological impacts will lay the foundation for developing microbiome-based strategies to enhance crop productivity and advance sustainable agriculture.

The advent of high-throughput sequencing (e.g., 16S/18S/ITS amplicon sequencing, metagenomics) has revolutionized our capacity to characterize microbial communities. However, microbiome data are compositional, sparse, and highly dimensional [13]. These limitations have prompted the use of artificial intelligence (AI), particularly machine learning (ML) and deep learning (DL), to extract meaningful patterns from complex, noisy datasets. The use of AI in human microbiome research has led to breakthroughs in handling these data challenges, giving rise to sophisticated methods for dimensionality reduction, feature selection, classification, and functional prediction [[14], [15], [16]].

Inspired by these advances, scientists are increasingly adapting these tools for rhizosphere research. AI is reshaping rhizosphere research by allowing researchers to move beyond taxonomic description to predictive modeling and functional inference. ML approaches are now used to identify key microbial biomarkers and predict community assembly outcomes [[17], [18], [19]]. Furthermore, AI is now being used to integrate microbiome data with host phenotypes, providing a new strategy for predictive breeding. For instance, features of the rhizosphere microbiome, such as operational taxonomic units (OTUs), amplicon sequence variants (ASVs), and taxonomic abundances, can serve as predictors of agronomic traits, in some cases outperforming models based solely on host genetics [[20], [21], [22], [23]]. This strategy, known as microbiome-enabled genomic selection (MGS), leverages microbial data alone for phenotypic prediction. Furthermore, the use of models that combine host SNPs with microbial markers significantly enhances the prediction accuracy for nutrient uptake and yield in crops such as maize and foxtail millet (*Setaria italica*) [24,25]. This approach, termed hologenome-enabled genomic selection (HGS), treats the microbiome as a dynamic, environmentally responsive extension of the host genotype, representing a novel strategy for the genetic improvement of crops by leveraging microbial community data to predict and enhance agronomic traits.

A key application of the hologenome concept is the rational design of synthetic microbial communities (SynComs). These engineered microbial consortia are tailored to enhance specific host traits, offering innovative solutions for generating climate-resilient crops [5]. As the field shifts from descriptive studies to functional applications, the empirical design of SynComs, whether by simplifying natural consortia or assembling defined isolates, has become a major bottleneck [26]. AI represents a transformative approach for addressing this issue via predictive design. AI can guide the assembly of functionally complementary strains, predict cross-feeding dynamics and consortium stability, and optimize multispecies compositions to enhance plant resilience [27,28]. Moreover, AI can predict emergent behaviors from complex interactions, minimizing reliance on trial-and-error [29]. This AI-driven strategy stands in sharp contrast to traditional methods. For example, whereas traditional methods successfully identified a low-nitrogen-enriched SynCom to promote soybean (*Glycine max*) growth under nitrogen stress [30], AI can accelerate this process by designing microbial consortia with defined functions in silico prior to empirical testing. This AI-driven strategy can lead to breakthroughs in the development of novel, climate-adaptive crops.

In this review, we examine advanced AI methods adapted from human microbiome studies to address key data challenges in investigating the rhizosphere microbiome (high dimensionality, sparsity, and compositionality), focusing on multimodal integration and language models, and assess the utility of these methods for functional prediction. We then explore AI-driven approaches, including the predictive modeling of rhizosphere interactions, integration of plant phenotypic and microbiome data, and design of synthetic microbial communities. Finally, we discuss current challenges and future directions for the use of AI in rhizosphere microbiome research. We propose an AI-optimized paradigm that integrates complementary inside-out (HGS) and outside-in (SynCom design) strategies, powered by transformative technologies such as federated learning (FL), large language models (LLMs), AI agents (or agentic AI), and digital twins (DTs). These advanced approaches will accelerate crop improvement and promote microbiome-driven sustainable agricultural development.

## 2 Learning from the human microbiome: AI for high-dimensional, sparse, compositional data

**Fig. 1:**
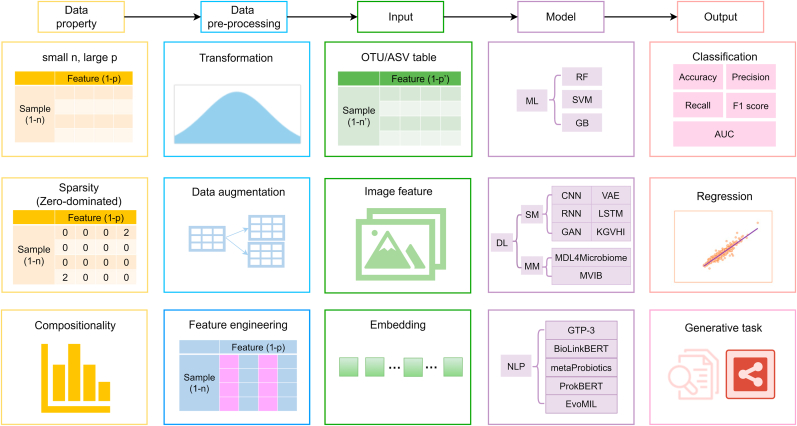
A generalized workflow for AI-driven analysis of microbiome data. The process begins with analyzing data properties, followed by pre-processing steps. The data are then used as input for various AI models to generate biological insights and predictions. The microbiome dataset is characterized by three key properties: high dimensionality (small sample size n and large feature number p), compositionality, and sparsity (zero-inflated). Data pre-processing typically involves three steps: transformation, augmentation, and feature engineering, yielding processed data in various formats such as OTU/ASV tables, image features, or embedding representations. Three primary approaches are employed for model training: machine learning (ML); deep learning (DL), including both single-modal (SM) and multimodal (MM) architectures; and natural language processing (NLP). These models generate outputs for diverse tasks including classification, regression, and generative modeling. OTU: operational taxonomic unit; ASV: amplicon sequence variant; RF: random forest; SVM: support vector machine; GB: gradient boosting; CNN: convolutional neural networks; RNN: recurrent neural networks; GAN: generative adversarial networks; VAE: variational autoencoders; LSTM: long short-term memory; MVIB: multimodal variational information bottleneck; GPT-3: generative pre-trained transformer 3; EvoMIL: evolutionary scale modeling with multiple instance learning; AUC: area under the curve. The figure was created in draw.io, with additional elements generated in PowerPoint and Excel.

Microbiome sequencing data presents unique analytical challenges due to its high dimensionality, sparsity, and compositionality (Fig. 1). High dimensionality is a feature of microbial datasets with a small n (limited samples) but large p (thousands of microbial taxa as features), leading to statistical instability and overfitting of ML models. Data sparsity stems from the prevalence of rare taxa (microbes present at low abundance or undetected in most samples), combined with technical limitations such as sequencing depth biases. Additionally, the compositional nature of microbiome data introduces fundamental constraints to ecological interpretation.

To address these challenges, researchers have developed innovative AI methodologies that are advancing human microbiome research. Dimensionality reduction techniques, such as principal coordinates analysis and t-distributed stochastic neighbor embedding, compress high-dimensional data into manageable embeddings, while interpretable tree-based models such as endoR [14] analyze microbial interactions based on decision rules and feature importance. For compositional data, KernelBiome uses specialized kernels to handle sparsity and compositionality while incorporating phylogenetic information [15]. In temporal studies, convolutional neural network-long short-term memory architectures with self-knowledge distillation capture longitudinal patterns effectively, achieving area under the curve (AUC) scores of 0.80–0.89 for clinical datasets [31]. Knowledge graph-based approaches like KGVHI integrate microbial networks with biological features using DL, demonstrating >95 ​% accuracy in predicting human–virus interactions [32]. As reviewed elsewhere, several other DL methods have been employed in human microbiome studies, including recurrent neural networks, variational autoencoders, and generative adversarial networks [33].

Multimodal learning, involving the integration of diverse data types to improve predictive performance, has emerged as a powerful method in computational biology. In microbiome research, combining taxonomic, genomic, and metabolic features can capture complex host–microbiome interactions that single-modality approaches may miss. For example, MDL4Microbiome is a multimodal DL framework that leverages taxonomic profiles, genome-level relative abundance, and metabolic functional features to significantly enhance disease prediction accuracy [34]. The strength of this method lies in the joint analysis of multi-dimensional microbiome signatures, offering insight into disease mechanisms. Multimodal variational information bottleneck (MVIB), representing a further advancement in the field, generates compact, informative joint representations from heterogeneous data modalities [35]. MVIB maintains competitive classification accuracy while improving computational efficiency, addressing a key limitation in large-scale microbiome analyses.

Recent advances in natural language processing (NLP) have revolutionized computational microbiology and virology using language-inspired models. Transformer-based architectures such as BioLinkBERT and GPT-3, fine-tuned on biomedical knowledge, were used to extract microbiome-disease relationships from the scientific literature, achieving high performance (F1, precision, recall >0.80) [36]. In genomic analysis, MetaProbiotics employs DNA sequence embeddings to analyze fragmented metagenomic data for probiotic discovery [37], while ProkBERT introduces efficient tokenization, i.e., encoding the sequence into a simpler vector format, for microbiome-specific tasks such as predicting promoters and phage genomes [38]. Another NLP-inspired model generates contextualized microbial embeddings to infer taxon interactions and functional roles within communities [39]. To predict virus–host interactions, protein language models combined with multiple instance learning achieve high accuracy (AUC >0.95) and identify key viral proteins using interpretable attention mechanisms [40]. These approaches benefit from self-supervised pretraining to mitigate data scarcity and use domain-adapted architectures for genomic sequences. However, challenges remain, including the limited availability of eukaryotic data and high computational costs. Embedding methods and graph models help reduce dimensionality and represent microbial co-occurrence networks. Promising directions include transfer learning and interpretable AI techniques such as Shapley additive explanations (SHAP) or local interpretable model-agnostic explanations for identifying key taxa or features. Integrating NLP with microbiome research helps address shared issues such as data sparsity, compositionality, and feature selection, opening new avenues in both fields.

## 3 From taxonomy to functional prediction: AI-driven insights into microbial traits

AI methods developed for human microbiota are moving beyond taxonomy to directly predicting the functional traits of microbes that underpin host health, including metabolite production and interaction mechanisms. These advanced capabilities are essential for the rational design of SynComs. A prime example is the BacterAI platform, which uses autonomous AI-driven experimental design to map microbial metabolic preferences without prior knowledge, surpassing the limitations of genomics-based functional inference [41]. Other studies have used coupled multilayer perceptrons to model personalized metabolic responses to dietary interventions. Through sensitivity analysis, these models can infer tripartite food–microbe–metabolite interactions, with predictions validated in synthetic datasets and supported by real-world findings in the literature [42]. In the area of biosynthetic gene cluster (BGC) prediction, several AI-driven advances are particularly notable. Hannigan et al. (2019) developed DeepBGC, a DL tool that uses a bidirectional long short-term memory network and NLP (pfam2vec embeddings) to improve BGC detection accuracy and reduce false positives [43]. DeepBGC can identify novel BGC classes and predict their products using RF classifiers [43]. To further refine BGC predictions, Almeida et al. (2022) introduced a reinforcement learning approach that optimizes BGC boundary identification and composition using Pfam domains and functional annotations [44]. This method improved the precision of gene prediction in fungal genomes by over 15 ​% and BGC prediction by over 25 ​% [44]. Mei (2023) proposed a multi-label learning framework that simultaneously predicts the chemical classes and biological activities of natural products directly from BGCs, eliminating the need for experimental structural resolution [45]. This approach exemplifies the type of AI-driven functional annotation that is critical for understanding plant–microbe interactions, particularly in the rhizosphere [45].

These technological breakthroughs have not only deepened our understanding of the human microbiome but have also given rise to a function-oriented research paradigm, demonstrating significant potential for use in other complex ecosystems such as the crop rhizosphere. The rhizosphere presents unique analytical challenges that mirror and extend those encountered in human microbial ecosystems: extreme dimensionality, pronounced sparsity, and strict compositionality constraints. Moreover, rhizosphere systems introduce additional layers of complexity due to tripartite plant–microbe–soil interactions and environmental modulations, which demand specialized analytical frameworks. Although not yet attempted, the AI methods and tools developed for human microbiota can be used directly or can be modified to analyze rhizosphere microbial data.

## 4 AI-driven analysis of rhizosphere microbiome assembly and dynamics via key taxa

A crucial application of AI in analyzing rhizosphere microbiome data is the identification of key taxa to help decipher microbial assembly and dynamics. ML approaches, particularly the random forest (RF) algorithm, have recently become indispensable for decoding complex rhizosphere dynamics. The consistent superiority of RF across studies, from identifying important bioindicator taxa [17,18] to revealing domestication-driven selection of rhizosphere taxa [46], points to an intrinsic alignment between the algorithm's hierarchical decision-making structure and the self-organizing nature of microbial communities. This intrinsic advantage of RF is highlighted by its exceptional performance in classifying plant-associated *Pseudomonas* strains. Specifically, RF successfully distinguished between beneficial (plant growth-promoting rhizobacteria) and pathogenic groups by analyzing genome properties, achieving remarkably high accuracy (AUC ​= ​0.99) [47]. Combining RF with microbial co-occurrence networks or gradient boosting methods further expanded its capabilities, revealing keystone taxa predictive of cadmium accumulation in rice (*Oryza sativa*) [17], bacterial growth rate as a driver of rhizosphere community assembly [19], and key rhizosphere bacterial/fungi genera to distinguish different groups of tea plant germplasm resources [48,49]. RF algorithms have become the dominant approach for feature selection and hypothesis generation due to their robust performance with sparse data and their ability to provide biologically interpretable results, such as ranking the importance of specific microbial taxa in driving a particular ecological function or plant phenotype.

The application of DL is particularly suited for scenarios where the relationships between microbes and environmental factors are highly non-linear and complex. DL has been successfully employed in microbiome research, as exemplified by the heterogeneous autoencoder framework [50] and the H2O-based (two hidden layers) neural network model [51], to overcome critical challenges in dimensionality reduction and data scarcity. By compressing high-dimensional microbial data into interpretable biomarkers while maintaining exceptional predictive power (AUC ​= ​1.0 or *r* ​> ​0.9), DL identified robust signatures of soil health and enhanced generalizability across ecological contexts. These achievements demonstrate the potential of DL to provide scalable, mechanistic insights into rhizosphere microbiome science, bridging gaps between theoretical models and real-world applications.

Despite these advances, critical challenges remain in translating computational insights from rhizosphere microbiome data into biological insights and practical applications. The current predominance of RF may reflect limitations in available datasets rather than true algorithmic superiority, while the significant performance gap between controlled and field conditions underscores the need for more environmentally contextual models. Researchers must prioritize developing hybrid architectures that combine interpretability with predictive power, establishing standardized evaluation frameworks, and integrating mechanistic models in order to integrate statistical patterns with the biological processes of rhizosphere microorganisms.

## 5 AI-driven integration of phenotypic and rhizosphere microbiome data

**Fig. 2:**
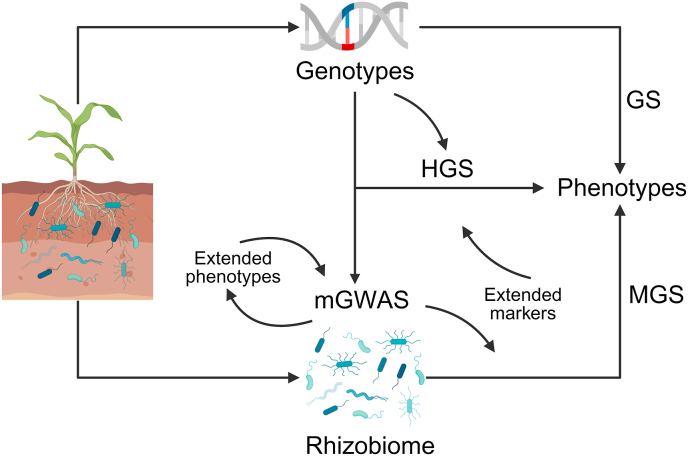
The complex interactions among crop genotypes, the rhizobiome, and phenotypes. Features of the rhizosphere microbiome (rhizobiome) can be conceptualized as extended phenotypes, linking them to crop genotypes through microbiome genome-wide association studies (mGWAS). The associations between rhizosphere microbiomes and phenotypes can be investigated using microbiome-enabled genomic selection (MGS), where operational taxonomic unit or amplicon sequence variant data serve as extended markers analogous to genomic markers in traditional genomic selection (GS). Hologenome-based genomic selection (HGS) integrates both rhizosphere microbiome markers and host genomic markers to improve phenotype prediction accuracy. This integrated approach ultimately enables the synchronous optimization of crop genotypes and their functionally associated rhizosphere microbiomes. The figure was created in BioRender.

In addition to identifying core taxa, a major application of AI is linking rhizosphere microbiome composition (including core microbes) to plant phenotypes for predictive modeling. The predictive power of microbial features has been demonstrated across crop systems, but the practical significance of these findings requires careful evaluation. For instance, although RF models using rhizosphere microbiota showed superior performance for predicting rice biomass and nitrogen accumulation [21], their actual improvement over conventional approaches might be marginal for agricultural applications. Similarly, their 84.6 ​% accuracy in classifying potato (*Solanum tuberosum*) yields [22] and near-perfect disease classification in citrus [23] represent promising but preliminary results that demand validation across diverse environments and seasons. By using microbes to predict plant phenotypes, the methods employed in these studies align with MGS. This innovative approach reconceptualizes the microbiome as a dynamic, environmentally responsive “extended genomic marker” that complements conventional host genetic markers in predictive models (Fig. 2). MGS offers unique advantages, including the ability to capture nuanced genotype-by-environment interactions through microbial community dynamics, access to direct functional annotations via metagenomic features, and the potential to monitor temporal trait variations through longitudinal profiling.

Building upon MGS, HGS incorporates both rhizosphere microbial markers and host genomic SNPs and has achieved higher predictive performance than conventional genomic selection (Fig. 2) [24,25]. This approach is particularly valuable for examining complex traits for which plant–microbe interactions play crucial roles, such as nutrient use efficiency, drought tolerance, and soil-borne disease resistance [7,52,53]. For example, by identifying crop germplasms with plant alleles and microbial taxa associated with nitrogen uptake, HGS models can provide hologenomic estimated breeding values. This allows breeders to simultaneously select for optimal plant genetics and the ability to recruit beneficial nitrogen-cycling microbes. This dual selection strategy represents a paradigm shift in crop improvement, as it allows breeders to develop cultivars that possess superior genetics while actively shaping their rhizosphere to enhance productivity. Such varieties would maintain more stable yields across varying soil conditions and require fewer agricultural inputs, representing a significant advance toward more sustainable cropping systems.

Despite the great promise of HGS, its practical application still faces multiple challenges. These include the computational complexity and lack of standardization in multi-omics data integration, the instability of microbial communities due to environmental sensitivity, the high costs associated with large-scale sequencing and analysis, the difficulty in translating statistical results into actionable biological mechanisms, and the modeling complications arising from the temporal dynamics of plant–microbe interactions. Together, these factors limit the broad application of HGS for practical crop breeding.

## 6 Engineering the rhizosphere: AI-guided design of synthetic microbial communities

Beyond AI-driven MGS/HGS, the AI-guided design of SynComs represents another crucial approach to leveraging rhizosphere microorganisms for the genetic improvement of crops, particularly for plant adaptation to climate change. Several integrated experimental-computational frameworks have emerged that incorporate AI-driven approaches into empirical SynCom construction to identify microbial combinations that enhance crop phenotypes. For example, Herrera et al. (2018) used neural networks to design rhizosphere microbial communities that alter plant phenotypes [54], while Emmenegger et al. (2023) used RF and an elastic-net regularized generalized linear model to develop pathogen-suppressing consortia [55]. While these studies successfully identified and validated key protective bacterial strains using synthetic communities and ML methods, it is important to note that their findings were obtained using simplified systems and may not readily translate to field conditions due to complex soil ecology and competition from indigenous microbiota.

Recent reviews have proposed iterative conceptual workflows for function-driven SynCom design that center on the important role of AI. These frameworks merge high-throughput functional screening with computational modeling to establish a closed-loop “design-build-test-learn” cycle [29]. The process begins with the construction of a functional trait matrix to inform SynCom design, followed by in silico simulation using genome-scale metabolic models and dynamic flux balance analysis to predict community interactions and dynamics [27,29]. Finally, ML leverages multi-omics and high-throughput phenotyping data to iteratively refine predictions, thereby continuously optimizing the SynComs for enhanced functionality, stability, and utility in the field [27,29].

Therefore, AI-guided design represents a fundamental transformation in SynCom research, transitioning from taxonomy-driven to prediction-driven assembly. However, this approach faces key challenges, including accurately predicting the functions of emergent communities, ensuring ecological persistence amid indigenous microbiota, and successfully scaling laboratory-optimized consortia for field applications. The continuous integration of trait-based design, multi-omics data, model-based simulation, and computational validation will expand our capacity to deploy effective rhizosphere microbial communities for crop improvement.

## 7 Key challenges and future directions

Unlocking the rhizosphere represents a critical frontier for advancing agricultural sustainability. Despite its potential, the application of AI to decipher this complex ecosystem remains critically underdeveloped compared to human microbiome research. In this section, we outline key bottlenecks in terms of data, algorithms, and model integration that currently limit progress in this area.

First, inadequate data quantity and quality pose a fundamental constraint. Rhizosphere studies typically feature smaller sample sizes, greater environmental variability, and sparser functional annotations than well-curated human microbiome projects. This data scarcity, exacerbated by the unpredictable influence of environmental factors on microbial communities, severely restricts model training and generalization. Second, there is a pervasive focus on microbial taxonomy over function. Although AI can identify taxonomic biomarkers, its ability to predict critical functional traits remains limited. This inability to forecast processes such as nutrient cycling or novel metabolite biosynthesis hinders the development of mechanism-based applications. Third, algorithmic simplicity persists, with most studies relying on traditional ML methods for microbial marker identification or crop phenotype prediction rather than adopting more sophisticated approaches commonly used in human microbiome studies such as neural networks, transformers, or graph-based models. Finally, integration across data types is rare. To date, only two published studies integrated rhizosphere microbial OTU/ASVs with genomic markers using traditional linear regression models for predicting important agronomic traits [24,25]. Rhizosphere research seldom combines microbial data with plant genomics, soil parameters, and climate variables into unified AI models, unlike the multi-omics integration routine in human studies. Technical hurdles further complicate this integration, such as data heterogeneity, high dimensionality, and complex interactions. These limitations collectively hinder the development of robust, high-precision AI tools for rhizosphere microbiome engineering and agricultural applications.

To advance the use of AI in rhizosphere microbiome research, three critical directions should be pursued. First, it is crucial to build standardized, large-scale databases. These databases must prioritize longitudinal data capturing microbiome dynamics in response to climate stressors such as drought and warming. Recent initiatives, such as the development of bacterial collections from the roots of crops and viral genome collections [56], are foundational; the critical next step is to enrich these with environmentally contextualized time-series databases. Second, the field must adopt more advanced AI architectures, such as transformer models and transfer learning, which are better suited to model complex plant–microbe–environment interactions and predict functional outcomes from multi-omics data. Finally, the development of multimodal approaches and explainable AI that integrate microbiome data with omics layers, agronomic variables, and comprehensive soil metadata using sophisticated DL frameworks will provide a more holistic understanding of rhizosphere systems. Compared to single-modal models, multimodal approaches remain underexplored but hold great promise. For instance, attention mechanisms can identify which microbial taxa exhibit coordinated variation with certain SNPs in functional genes in the host plant related to root architecture or nutrient uptake, while explainable methods such as SHAP values provide a rigorous framework to quantify the marginal contributions of both host genetic and microbial features to phenotypic outcomes. Such integrative frameworks could improve predictive accuracy and uncover biologically meaningful interactions across data layers. These advancements could help bridge the gap between AI applications for human and crop rhizosphere microbiome research.

## 8 Emerging paradigms toward an intelligent and integrated future for hologenome-assisted breeding

**Fig. 3:**
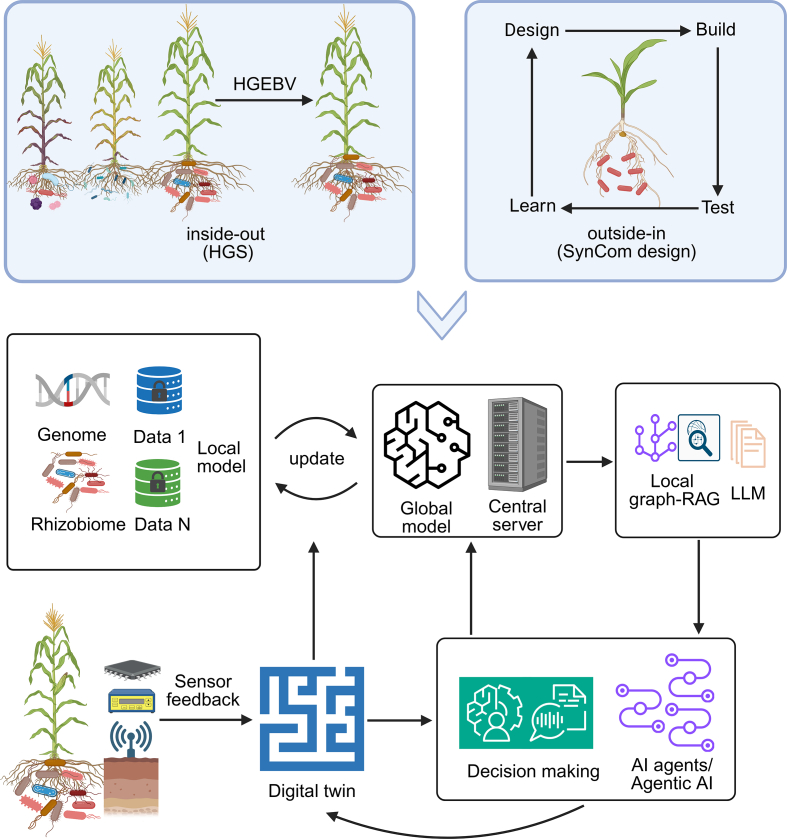
A synergistic inside-out/outside-in framework for hologenome-assisted breeding powered by core AI technologies. The core of this framework lies in the integration of two complementary approaches: hologenome-based genomic selection (HGS) and synthetic microbiome design (SynCom). Four core AI technologies help the framework form a closed-loop learning system. Federated learning (FL) serves as the privacy-preserving data backbone, enabling the collaborative training of models across distributed institutions without sharing raw multi-omics data. A central server aggregates local model updates to build a robust global model. Large language models (LLMs), enhanced by knowledge graphs and retrieval-augmented generation (RAG) from the scientific literature, act as a knowledge engine to inform predictions. Digital twins (DTs) simulate the agro-ecosystem in real-time using IoT data, providing a virtual space to validate and optimize plant genotype and SynCom combinations. Finally, autonomous AI agents orchestrate the entire pipeline. These agents manage DT simulations, analyze outcomes, and route feedback; fundamental model discrepancies trigger global model retraining through the FL server, while time-critical operational anomalies prompt immediate, localized interventions. This creates a system that continuously refines both global intelligence and local actions. HGEBV: hologenomic estimated breeding value. The figure was created in BioRender, incorporating elements initially generated in draw.io.

Modern agricultural practices must address three critical challenges: climate change-induced environmental stress [57], escalating soil degradation [58], and the urgent need for ecologically sustainable crop improvement strategies [4,6]. Traditional breeding approaches often overlook the critical role of the plant-associated microbiome. However, recent advances are paving the way for microbiome-assisted improvement, from identifying host genetic loci that enhance beneficial microbiome recruitment to the concept of hologenome breeding, which selects for optimal plant–microbe holobionts [59]. Here, we present a framework that synergistically integrates an AI-powered inside-out approach using HGS to develop plant genotypes that recruit beneficial microbiota with an outside-in approach using SynCom design to construct tailored microbial communities (Fig. 3). This AI-assisted framework could be utilized for the rapid development of tailored inoculants and their optimal deployment with elite crop varieties under specific environmental and temporal conditions [59].

This integrated pipeline of FL, LLMs, DT, and autonomous agents effectively operationalizes the synergistic inside-out/outside-in framework. FL serves as the privacy-preserving data backbone of this framework, enabling the collaborative training of predictive models for HGS and SynCom design across distributed institutions and farms without the need to share raw multi-omics data. This is crucial for building robust models that require diverse environmental validation. Recent advances are overcoming key implementation barriers: Wang et al. (2024) developed FedType to aggregate heterogeneous multi-omics data [60], while Tuncel and Öztoprak (2025) integrated FL with blockchain to secure IoT-enabled edge devices for agricultural monitoring [61].

LLMs act as the knowledge engine for this integrated pipeline. While LLMs have advanced human microbiome research, their application in agriculture is only just emerging, with models such as SeedLLM [62] for rice and PlantGPT [63] for *Arabidopsis thaliana*. These tools can extract rhizosphere microbial–crop interaction patterns from the scientific literature, generating hypotheses and advising on SynCom formulations. However, a dedicated LLM for the rhizosphere microbiome remains a critical unmet need to fully leverage this potential. The newly designed crop genotype and SynCom combinations are then validated and optimized within a DT of the agro-ecosystem. The DT leverages real-time data from IoT sensor networks (e.g., portable microbial chips, SensorTalk/AgriTalk [64]) to simulate soil properties and rhizosphere dynamics. Crucially, the DT addresses the challenge of data scarcity for model training [65], as it generates rich simulation data that can, in turn, be used to refine the global models through FL, creating a learning cycle.

Autonomous AI agents (or agentic AI) orchestrate this entire pipeline, enabling real-time, closed-loop decision-making. Inspired by systems like Coscientist [66] and BehaveAgent [67], these agents coordinate the DT simulations. Autonomous AI agents analyze outcomes to determine the optimal feedback path: fundamental model discrepancies trigger retraining through the FL server for global improvement, while time-critical operational anomalies prompt immediate agent intervention for localized adaptation. Their decision-making is contextualized by LLM-powered knowledge graphs, which allow the agents to dynamically balance competing holobiont objectives, such as nutrient acquisition, pathogen suppression, and abiotic-stress tolerance.

**Fig. 4:**
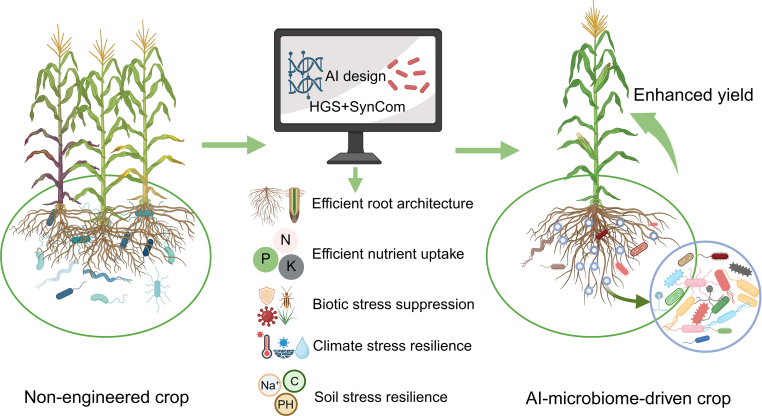
An AI-microbiome-driven crop. This AI-microbiome-driven maize plant is characterized by a precisely engineered root system. The root architecture is optimized for maximal soil exploration with minimal biomass investment via hologenome-based genomic selection (HGS). Additionally, the root surface is protected by a gelatinous “probiotic shield” biofilm comprising AI-designed synthetic microbial communities (SynComs). The crop demonstrates high nutrient absorption efficiency, enhanced tolerance to biotic and abiotic stress, and high carbon utilization efficiency, resulting in high, stable yields under a wide range of environmental conditions. While demonstrated here in maize, this framework is applicable to a wide range of crops. In the schematic, the blue circle on the root represents the biofilm, within which the different colored rhizosphere microbes denote distinct functional phenotypes. The figure was created in BioRender.

The AI-driven design of the rhizosphere microbiome represents a paradigm shift in sustainable agriculture. Our proposed approach synergistically combines HGS-screened plant genotypes with AI-optimized SynComs to create a novel crop system (Fig. 4). Such a crop would feature a precisely engineered root architecture that maximizes soil exploration, along with a root surface coated by a SynCom-derived “probiotic shield”. This crop would exhibit high nutrient absorption efficiency, enhanced resilience to both biotic and abiotic stress, and improved carbon utilization efficiency, which collectively contribute to high and stable yields across diverse environments. While this framework holds great promise, key challenges persist, including decoding hologenome integration mechanisms, handling spatial-temporal heterogeneity of the microbiome, and validating these simulations in real-world settings. Ultimately, by enabling targeted manipulation of the plant–microbe holobiont, this strategy will enhance climate resilience while reducing chemical inputs, directly addressing critical ecological and food security challenges.

## CRediT authorship contribution statement

**Juan Ma:** Writing – review & editing, Writing – original draft, Funding acquisition, Conceptualization. **Jiangfang Qiao:** Writing – review & editing. **Yanyong Cao:** Writing – review & editing. **Zeqiang Cheng:** Writing – review & editing.

## Declaration of competing interest

The authors declare that they have no known competing financial interests or personal relationships that could have appeared to influence the work reported in this paper.

## Data Availability

Data sharing is not applicable to this article as no datasets were generated or analyzed during the current study.
